# On the Occurrence of Fever at Cowbridge in 1853

**Published:** 1856-10

**Authors:** William Camps

**Affiliations:** one of the Foreign and Colonial Secretaries of the Epidemiological Society of London.


### ON THE OCCURRENCE OF FEVER AT COWBRIDGE, GLAMORGANSHIRE, IN THE AUTUMN OF 1853.

By WILLIAM CAMPS, M.D.; one of the Foreign and Colonial Secretaries of the Epidemiological Society of London.

(*Read before the Epidemiological Society*, *April* 2, 1855.)

TOWARDS the end of the year 1854, in consequence of an outbreak of cholera at Bridgend, in Glamorganshire, I visited that place by order of the General Board of Health. Whilst I was there my attention was, on several occasions, directed to the occurrence of a fever in the autumn of the year preceding at Cowbridge, in the same county, a small town situated at the distance of seven or eight miles from Bridgend, and constituting a part of the same union, under the name of the Bridgend and Cowbridge union. There are several interesting as well as remarkable features connected with this outbreak of fever; and as there exists at present, to my knowledge, no authenticated record of this epidemic, drawn up by medical authority, I have been requested by Dr. Gavin Milroy to present this notice of that epidemic to the Epidemiological Society.

Cowbridge is a small town, situated between Bridgend and Cardiff, eastward of the former place, although not on the line of railroad connecting those two places, but on the road still in use for horse and carriage conveyance. Its population does not greatly exceed one thousand persons.

As in many other towns, horse races are held at Cowbridge at regular periods; and at the races held here, it has been the custom for a long time for the gentry of the surrounding neighbourhood to assemble together at one or more balls, during what is termed the race week, which is thus made a season of unusual gaiety and festivity for the inhabitants of the place and its adjacent neighbourhood. I have been told that these races, with their accompanying balls, are, for the most part, held at the early part of the month of November. In the year 1853, in that month, and during the race week, there were two balls, separated by one intervening night; and it was immediately after these two balls that many of those who were present at them were seized with symptoms of alarming disorder, which, in not a few cases, terminated fatally. So far as I could learn from repeated inquiries whilst in the neighbourhood, and from various persons, some of whom had formed part of the gay assembly, this disorder assumed the character of a very malignant fever.

Among the striking particulars connected with the Cowbridge fever, it was especially observed that no matter from what part of the country those came who attended these balls, or to what part they returned, nearly all suffered afterwards, and some died of a disorder described as fever. Thus, I was informed that some members of different families returned to Devonshire or Somersetshire, some to one of the midland counties, to Yorkshire, and, I think it was stated (but of this I cannot be positive), that one or more returned to Scotland from Cowbridge; of these, most fell ill, and suffered from long and severe indisposition, attended with extreme depression and prostration of strength, of which two or three died. These balls were held on this occasion, as I believe is customary at such towns, at the chief inn in the place, and in rooms partially, if not altogether, fitted up as was then required. It will be seen from the following extract from the report of the Inspector of Nuisances of the town of Cowbridge, that the supper room was a temporary erection; and previously to the ball, it was a loft over a seven-stalled stable. The inspector, in his report, under head No. 39, remarks as follows:—

"I inspected the Bear Inn premises, and could not find any drain that had been recently opened. There is one water closet in the house supplied with water, which flushes the contents through the various drains connected with the house, and from thence to the town ditch. I found the passage connecting the ball room with the supper room had been recently erected with boards covered with paper; this passage was partly built over a tank of water, which drains from the roof of the premises, and is used for house purposes. The supper room, previously to the ball, was a loft over a seven-stalled stable; the ceiling of the supper room was constructed of boards covered with white calico. The supper room appeared to have been recently papered; the walls were dry, and the plaster seemed to be of several years standing. I examined the flooring of the supper room, and found no steam could arise from the stable underneath. I examined the stable under the supper room, and found nothing but surface drainage there. There was an offensive smell arising from a privy in the passage leading from the stable to the house. The landlord stated that the smell was caused by the privy being emptied the morning of my inspection, which I afterwards found to be true. There seems to be a good supply of water from the tank, and a pump which flushes all the refuse through the drains leading from the house into the town ditch. The landlady is now ill in bed with fever."

As might be reasonably expected, there are some discrepancies in the statements made by different persons, in regard to the number of individuals of both sexes who were present at these balls, as well as to the number of those who became ill afterwards, and even of those who died from the disease with which they were attacked. Yet, in the main, there is a tolerably good amount of agreement between them all; so that the various statements are in nowise invalidated by each other. It must be remembered, that twelve months had elapsed from the time of the occurrence of this outbreak of fever to that at which I visited the neighbourhood; and by a singular coincidence, I was there, even in the town itself, on the anniversary of these races and balls, a period not unlikely to revive in many minds the lamentable occurrence of the preceding year. I was on many occasions in the company of persons, of both sexes, who had been present and had taken part in the amusement; one of these was a very intelligent medical practitioner residing in the neighbourhood, who had, in the discharge of his professional duties, been called upon to attend many of those who were attacked. I saw the sister of one who died; and another lady who was present, and who was attacked with the fever, from which she was long in recovering. The medical gentleman, already referred to as having been in attendance on several of the sufferers, as well as all other authorities from whom I could gain information respecting this disease, described it as typhus fever of a very low, malignant type, and characterised by symptoms of extreme debility and prostration of strength, requiring tonics and stimulants, and in many cases, more especially the fatal ones, accompanied by hæmorrhage from the bowels. One authority stated, that nearly one hundred and forty persons attended these balls; others told me the number present did not amount to more than one hundred persons. At the distance of time from the occurrence of the outbreak of the epidemic, when I was thereabouts, it was by no means easy to procure exact statistics as to those who were present, those who were taken ill afterwards, those who recovered, and those who died; but that several persons of both sexes succumbed under the attack, there can be no doubt; and from the evidence that I was able to collect, it may be fairly inferred that, at least in six or eight instances, if not more, this fever proved fatal, and that too, be it remembered, in a class of persons, who, when ill, from whatever cause, could and would secure for themselves all that medical skill, combined with domestic and hygienic accessories, might be expected to accomplish, towards restoration from the malady under which they suffered.

